# A Tale of Two Pathways: Tamsulosin MET Versus Historical Analgesic-Only Therapy for Pediatric Distal Ureteral Stones

**DOI:** 10.3390/healthcare14213632

**Published:** 2026-10-26

**Authors:** Predrag Ilic, Sava Cvetinovic, Marko Marjanovic, Ana Cvetinovic, Vladimir Kojovic

**Affiliations:** 1 Institute for Mother and Child Health Care of Serbia, 11000 Belgrade, Serbia; sava.cvetinovic@gmail.com (S.C.); marjanovicmdr@gmail.com (M.M.); drana.cvetinovic@gmail.com (A.C.); kojovic.dr@gmail.com (V.K.); 2 School of Medicine, University of Belgrade, 11000 Belgrade, Serbia

**Keywords:** pediatric urolithiasis, ureteral stones, distal ureter, medical expulsive therapy, tamsulosin

## Abstract

**Highlights:**

**What are the main findings?**

- Tamsulosin-based MET was associated with a higher observed spontaneous passage rate for 4–10 mm distal ureteral stones than historical analgesics alone (78.5% vs. 49.6%; risk difference 28.9 percentage points, 95% CI 20.7–36.6).
- Surgery was needed in 21.5% of children on MET vs. 50.4% on analgesics alone, also after adjustment for stone size and hydronephrosis. Passage was higher with MET in every age group (71–81% vs. 47–53%), with no significant age difference in effect.

**What are the implications of the main findings?**

- This single-center real-world experience is compatible with MET reducing operative management for distal ureteral stones up to 10 mm.
- A structured 3-week pathway with ultrasound reassessment at days 7/14/21 may provide a pragmatic framework for conservative management and timely intervention if needed.

**Abstract:**

**Background/Objectives**: Distal ureteral stones in children can cause severe colic and may lead to persistent obstruction potentially resulting in renal damage if not treated accordingly. This study assessed whether tamsulosin-based MET is associated with higher spontaneous passage of 4–10 mm distal ureteral stones and a lower need for surgery compared with analgesic therapy alone. **Methods**: In this single-center prospective observational cohort study with a historical comparator, 270 patients aged 5–18 years presenting with renal/ureteral colic and imaging-confirmed distal ureteral stones (4–10 mm) received tamsulosin-based MET (0.4 mg daily) from 2016 to 2023 and were stratified into three age groups. Follow-up included ultrasonography at days 7, 14, and 21. Outcomes were compared with a historical control cohort (2009–2015), involving 242 patients, treated with analgesics alone under the same criteria, follow-up and indications for surgery. The primary outcome was confirmed spontaneous stone passage during the 21-day conservative pathway, before any decision to operate; secondary outcomes included surgery (decision to proceed to ureteroscopy/laser lithotripsy by day 21) and its timing. **Results**: Spontaneous passage occurred in 78.5% of patients on MET versus 49.6% on analgesics alone (risk difference 28.9 percentage points, 95% confidence interval [CI] 20.7–36.6; *p* < 0.001), also after adjustment for stone size and hydronephrosis (adjusted odds ratio 4.52, 95% CI 2.95–6.91). Passage was higher with MET in all age groups (exploratory; treatment-by-age interaction *p* = 0.390), and surgery was decided in 21.5% versus 50.4%. No adverse effects were reported by parents, but they were not systematically recorded. **Conclusions**: Tamsulosin-based management was associated with a higher observed rate of spontaneous passage and a lower proportion undergoing ureteroscopy than historical analgesic-only care. These findings require cautious interpretation because of the non-contemporaneous comparison and potential confounding by calendar period, stone characteristics and intervention practices.

## 1. Introduction

Over the past two decades, the incidence of urolithiasis in the pediatric population has increased significantly, particularly among adolescents. Its geographical distribution varies according to climate, regional diet and other local epidemiological factors [1,2,3,4]. The increasing burden associated with this rise in incidence has led pediatric urolithiasis to be recognized as an emerging health problem rather than an occasional clinical finding. Given that inflammatory conditions and congenital anomalies are often first thought of in children, and urolithiasis is not taken into account, it is not so rare that the diagnosis of stones in the urinary tract is misdiagnosed or is an incidental finding during an ultrasound examination or radiography [2,3].

Symptomatic ureteral stones represent a particular clinical challenge due to pain, vomiting, and the development of obstruction of the renal collecting system, with a variable spectrum of clinical presentation. The treatment approach requires a balance between the likelihood of spontaneous stone passage and the risk of symptom progression and renal obstruction [5,6]. Regardless of whether the patient is an adult or a child, the evidence shows a strong correlation between spontaneous stone passage and both stone size and stone location. Smaller stones and stones located more distally have a higher likelihood of spontaneous passage, whereas increasing stone diameter is associated with a progressively lower probability of uncomplicated passage [5,6,7,8]. Clinical treatment protocols pay particular attention to the management of uncomplicated distal ureteral calculi of borderline size, where the greatest uncertainty exists regarding the timing of surgical intervention and the effects of a “watchful waiting” approach [5,6].

Medical expulsive therapy (MET), which involves the use of alpha-adrenergic blockers, is an established practice in the conservative management of ureteral calculi in adults [5,9,10,11,12]. The biological rationale for this therapy is based on the presence of alpha-1 adrenergic receptors in the smooth muscle of the ureter. The action of alpha-blockers leads to relaxation of spasms in the distal ureter and reduces resistance to stone passage, thereby promoting anterograde stone propulsion [9,10,11,13]. This mechanism provides the pharmacological basis for the use of tamsulosin as MET in adult patients with ureteral stones [9,10,11].

The evidence for MET in adults is, however, not uniform. Two large placebo-controlled trials, SUSPEND and STONE, did not find a significant increase in stone passage with tamsulosin [14,15]. In contrast, a large multicenter placebo-controlled trial and an Australian trial reported a benefit that was confined to larger stones, above 5 mm in the former [16] and 5–10 mm in the latter [12], and a meta-analysis of 55 randomized trials likewise suggested that the effect depends on stone size [10]. Stone size therefore appears to determine which patients are likely to benefit, and the 4–10 mm range studied here spans this threshold.

The adoption of this conservative approach in the pediatric population has been slow. Early prospective randomized trials and multi-institutional cohort studies conducted in pediatric patients have shown that tamsulosin may facilitate spontaneous passage of distal ureteral calculi [17,18]. Subsequent systematic reviews and meta-analyses have supported the use of MET in children, emphasizing higher rates of spontaneous stone expulsion and shorter expulsion times, while also highlighting the smaller number of trials and the more limited evidence base compared with the adult population [5,19,20,21,22].

These limitations remain clinically relevant, as the integration of MET into pediatric practice is still ongoing despite encouraging published studies [23]. When the available studies and clinical practice guidelines are considered, it can be concluded that the subgroup of patients with distal ureteral stones measuring 4–10 mm is clinically important, because spontaneous passage cannot be assumed, while surgical treatment may still be avoided [5,6,7,8,17,18,19,20,21,22]. Accordingly, this prospective observational cohort study with a historical comparator aimed to evaluate whether tamsulosin-based MET is associated with higher spontaneous passage of distal ureteral stones measuring 4–10 mm and a lower need for ureteroscopy and lithotripsy in children compared with analgesic therapy alone, and to explore whether this association differed by stone size, hydronephrosis and age. The study is intended to provide additional real-world single-center data that complement, and do not replace, randomized evidence.

## 2. Materials and Methods

### 2.1. Study Design and Setting

This was a single-center prospective observational cohort study with a historical comparator, conducted in the emergency care unit of the Institute for Mother and Child Health Care of Serbia. Consecutive eligible patients were identified in two cohorts: a prospective cohort treated with tamsulosin-based medical expulsive therapy (MET) from 2016 to 2023, and a historical cohort treated with analgesics alone from 2009 to 2015, whose data were retrieved retrospectively from the hospital medical records. Data for both cohorts were extracted from the hospital patient records, which remain available for verification. Tamsulosin was introduced into the institutional treatment protocol in 2016, and no other change to the management protocol was made. Treatment followed routine clinical practice and was not assigned by a study protocol, all eligible patients treated from 2016 received tamsulosin, and no legal guardian declined it. Each record represents a unique case of a distal ureteral stone, and no formal sample size calculation was performed. The study is reported following the STROBE recommendations.

### 2.2. Patients

Patients aged 5–18 years with renal/ureteral colic and an imaging-confirmed distal ureteral stone of 4–10 mm were eligible. The distal ureter was defined as the segment below the sacroiliac joint. Diagnosis was confirmed by ultrasonography, kidney–ureter–bladder radiography, or computed tomography, and stone size was measured on ultrasonography by a radiologist. Patients in both cohorts were selected according to the same criteria. Exclusion criteria were multiple stones, stones located in the middle or proximal ureter, congenital anomalies of the urinary tract, previous ureteral surgery, and severe hydronephrosis. Patients requiring urgent intervention, in whom conservative treatment was not appropriate, were not included in the study, such as those with urinary tract infection complicated by obstructive pyelonephritis, impending urosepsis, anuria, or a ureteral stone in a solitary kidney. Hydronephrosis was graded on ultrasonography using the Society for Fetal Urology (SFU) grading system and categorized as absent/mild (grades 0–1) or moderate (grades 2–3); grade 4 (severe) hydronephrosis was an exclusion criterion. Patients were stratified into three age groups (5–10, 11–14 and 15–18 years). The age groups were chosen for descriptive purposes. Age was recorded in whole years and stone size in whole millimeters. Body weight was not available for this analysis, laterality, renal function and symptom duration were not recorded and the date and reason for surgery were not extracted for this study.

### 2.3. Treatment

In both cohorts, patients received ibuprofen 10 mg/kg per dose as needed at intervals of at least 6–8 h, with a maximum of three doses per day (four doses in patients weighing more than 30 kg), and were advised to maintain hydration. If pain was not controlled with ibuprofen, the patient was hospitalized for observation and intravenous analgesia, and if this was not effective, surgical treatment was decided. Patients in the MET cohort additionally received tamsulosin 0.4 mg once daily. Because this use is off-label, legal guardians were informed, and the dose was based on pediatric studies published before MET was introduced into our practice [17,18]. Conservative treatment was continued until stone passage was confirmed or for a maximum of 21 days, in line with institutional practice. Day 1 was the start of treatment after confirmation of the diagnosis.

### 2.4. Follow-Up, Outcomes and Surgical Criteria

The same follow-up protocol was applied in both cohorts: clinical assessment and ultrasonography on days 7, 14 and 21, with earlier assessment in case of clinical deterioration. The decision to proceed to ureteroscopy with laser lithotripsy under general anesthesia was made at any time during follow-up for worsening symptoms, impaired renal function, signs of infection, or pain that could not be controlled with analgesics, and otherwise when the stone persisted at the end of the follow-up period the procedure was then performed in the following days and not as an emergency. Stenting was selective, according to intraoperative findings. Only endoureteral procedures are performed at our institution, and no spontaneous stone passage was documented between the decision to operate and ureteroscopy. Patients in whom surgery was decided were analyzed as not having achieved spontaneous passage.

The primary outcome was confirmed spontaneous stone passage during the 21-day conservative management pathway, before a decision to proceed to ureteroscopy, defined as the absence of symptoms and of the stone on follow-up ultrasonography. For patients requiring surgery, the decision to operate marked the end of conservative management. If an ultrasound finding was unclear, a repeat ultrasound examination was performed according to institutional protocol. Weeks of follow-up were defined as days 1–7, 8–14 and 15–21 from the start of treatment. Secondary outcomes were surgery, defined as the decision to proceed to ureteroscopy with laser lithotripsy by day 21 (failure of conservative management), and the week of follow-up (1, 2 or 3) in which this decision was made. Passage and surgery are complementary outcomes. Parents were asked about possible adverse effects during routine follow-up visits, but these observations were not systematically recorded, therefore, a formal safety analysis could not be performed. Treatment-related adverse effects were not prespecified as a secondary outcome in the original study protocol. Events during the treatment pathway, such as febrile infection, worsening obstruction, unplanned admission or urgent drainage, were not systematically recorded.

### 2.5. Ethical Considerations

This study was conducted in accordance with the principles of the Declaration of Helsinki. The study protocol was reviewed and approved by the Ethics Committee of the Institute of Mother and Child Healthcare “Dr Vukan Čupić”.

Prospective MET cohort (2016–2023): Consent for treatment and consent for participation in the study, including the use of study data, were obtained in writing on separate institutional forms. For patients younger than 15 years, both forms were signed by a parent or legal guardian. Patients aged 15–17 years signed the forms themselves together with a parent or legal guardian, and patients aged 18 years signed them independently. All patients received information about the proposed treatment and study procedures appropriate to their age and level of understanding; for younger children, no separate assent form was used, but their willingness to participate was considered.

Historical cohort (2009–2015): Data were obtained retrospectively from existing medical records and analyzed in anonymized form. The Ethics Committee approved this use of the records and did not require additional consent.

### 2.6. Statistical Analysis

Categorical variables were summarized as frequencies and percentages and compared using Pearson’s chi-square test or Fisher’s exact test, as appropriate. For the primary outcome, between-group differences were expressed as risk differences (RDs), risk ratios (RRs), and odds ratios (ORs), with corresponding 95% confidence intervals (CIs).

Multivariable logistic regression analysis was used to evaluate the association between treatment and spontaneous stone passage, with adjustment for relevant clinical variables in the respective models. Adjusted effects are presented as odds ratios (aORs) with 95% CIs. Potential effect modification by age, stone size, and hydronephrosis was assessed using interaction analyses.

Interactions were assessed on the odds-ratio scale using likelihood-ratio tests comparing nested models: treatment by age group in the model with treatment and age group; treatment by stone-size category in the model with treatment and stone-size category (without hydronephrosis) and, separately, in the model that also included hydronephrosis; and the two-way and three-way interactions involving treatment, stone-size category and hydronephrosis in the joint model.

All analyses were performed in patients with documented outcomes, patients lost to follow-up were excluded because their outcomes could not be determined. Sensitivity analysis was performed by comparing the three-year periods immediately before and after the introduction of MET (2013–2015 vs. 2016–2018). Subgroup and interaction analyses were considered exploratory, and Holm adjustment was applied to the three stone-size subgroup comparisons. All statistical tests were two-sided, and *p* < 0.05 was considered statistically significant. Statistical analyses were performed using Python version 3.13.5 with NumPy version 2.3.5 and SciPy version 1.17.0.

## 3. Results

The primary analysis compared the cohorts according to confirmed spontaneous stone passage during the 21-day conservative pathway, before any decision to operate, in patients with documented outcomes. All other analyses (stone size, hydronephrosis, age, interaction tests, adjusted models, calendar-window sensitivity analysis and timing of surgery) were secondary or exploratory and are identified as such. Effect measures are the risk difference (RD, in percentage points [pp], MET minus analgesic-only), the risk ratio (RR) and the odds ratio (OR), with the analgesic-only cohort as the reference and 95% confidence intervals (CIs); the methods are described in the Statistical Analysis subsection. Because every patient was classified as having either spontaneous passage or surgery, these two outcomes are complementary descriptions of the same distribution and not independent evidence of efficacy.

### 3.1. Patient Flow and Study Population

**Figure 1:**
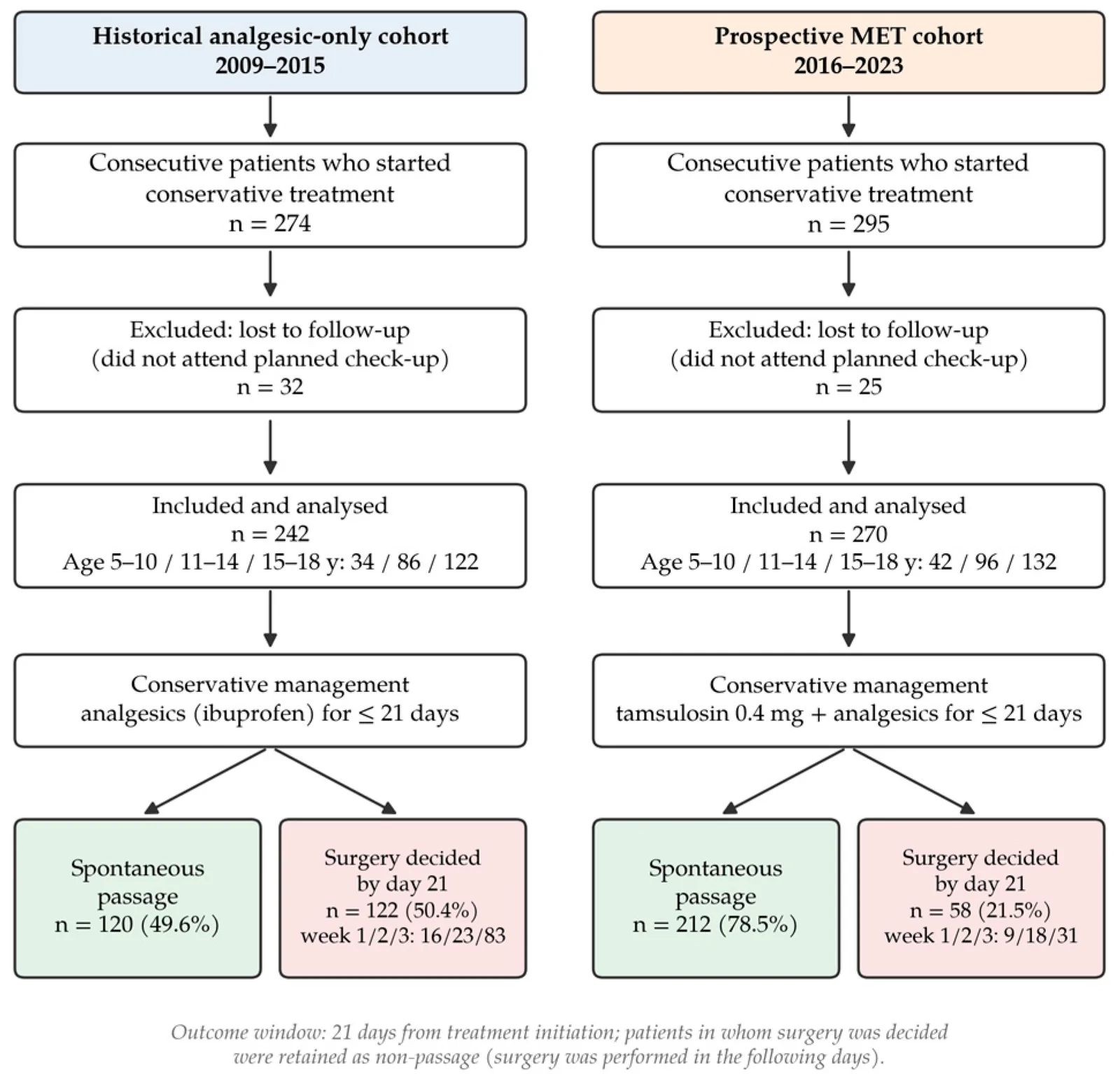
Flow of patients through the study. The historical analgesic-only cohort (2009–2015) and the prospective tamsulosin-based MET cohort (2016–2023) were observed for up to 21 days from treatment initiation, and all included patients were analyzed irrespective of the timing of the decision to operate; surgery was performed in the days following the decision in all 180 patients. Patients lost to follow-up were excluded because their outcomes could not be determined. The number of patients screened was not recorded. MET, medical expulsive therapy.

A total of 569 patients with a distal ureteral stone of 4–10 mm initiated conservative treatment: 274 in the historical analgesic-only cohort (2009–2015) and 295 in the prospective MET cohort (2016–2023). Of these, 57 patients (32 analgesic-only [11.7%] and 25 MET [8.5%]) did not attend scheduled follow-up examinations and were excluded from all subsequent analyses because their outcomes could not be determined. The final analysis included 512 patients with documented outcomes (242 analgesic-only and 270 MET), to whom all reported passage and surgery proportions refer (Figure 1). The number of patients screened was not recorded. All 512 analyzed patients were assigned to one of two outcome categories within the 21-day observation period (spontaneous passage or a decision to proceed to ureteroscopy with laser lithotripsy by day 21), patients in whom surgery was decided were counted as not having achieved spontaneous passage. All 180 patients in whom ureteroscopy was decided subsequently underwent the procedure, and no spontaneous stone passage was documented between the surgical decision and the intervention.

### 3.2. Baseline Characteristics

Baseline characteristics are summarized in Table 1. The cohorts did not differ in age-group distribution (*p* = 0.880), sex (45.2% vs. 43.4% girls; *p* = 0.683), stone-size category (*p* = 0.952) or frequency of moderate hydronephrosis (28.9% vs. 29.3%; *p* = 0.911). Median age was 14 years (IQR 12–17) in the MET cohort and 15 years (IQR 12–17) in the analgesic-only cohort (*p* = 0.907), and median stone size was 6 mm (IQR 5–7) in both cohorts (*p* = 0.802). In both cohorts, stones of >5–7 mm were the most frequent (47.4% and 47.9%), and about one fifth of stones measured >7–10 mm (20.4% and 21.1%). In the three-year windows adjacent to the introduction of MET (analgesic-only 2013–2015; MET 2016–2018), baseline characteristics were likewise similar (all *p* ≥ 0.333; Table 1, lower panel).

### 3.3. Primary Outcome: Spontaneous Stone Passage

**Figure 2:**
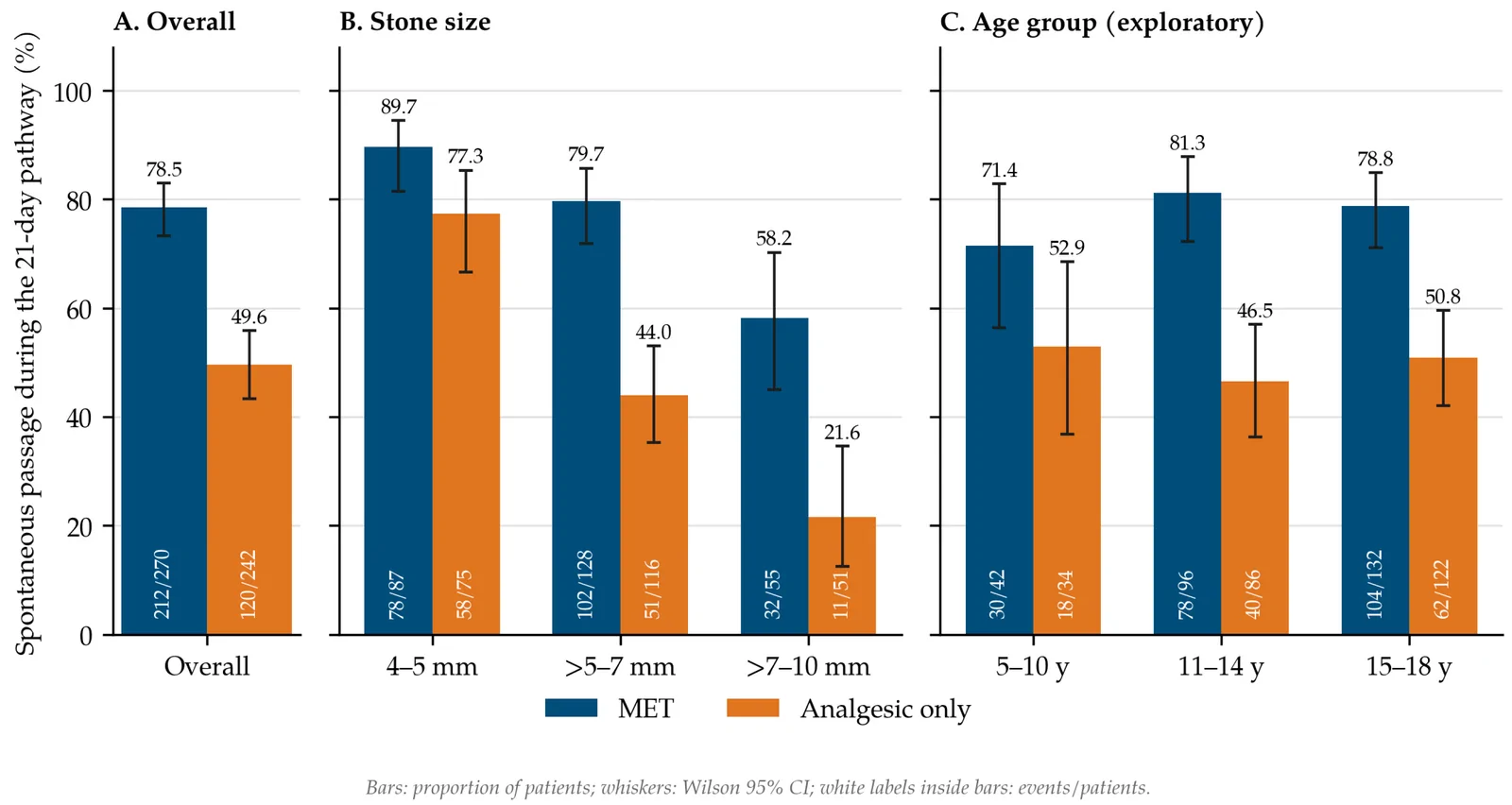
**Confirmed spontaneous stone passage during the 21-day pathway** in the MET cohort (*n* = 270) and the analgesic-only cohort (*n* = 242): (**A**) overall (primary analysis); (**B**) by stone-size category; (**C**) by age group (exploratory). Bars show the observed proportion, whiskers the Wilson 95% CI, and the numbers inside the bars the events/patients. The cohorts were treated in different calendar periods (see Section 3.5 and Section 3.7).

Confirmed spontaneous passage during the 21-day, occurred in 212 of 270 patients (78.5%; 95% CI 73.2–83.0%) in the MET cohort and in 120 of 242 patients (49.6%; 95% CI 43.3–55.8%) in the analgesic-only cohort (Table 2, Figure 2A). The absolute difference was 28.9 pp (95% CI 20.7–36.6), and the risk ratio was 1.58 (95% CI 1.37–1.82), corresponding to a 58% higher relative probability of passage in the MET cohort. The odds ratio was 3.72 (95% CI 2.53–5.46); because passage was common in both cohorts, the OR is considerably larger than the RR and must not be read as a 3.7-fold higher probability. The difference was statistically significant (Pearson χ^2^(1) = 46.9, *p* < 0.001; Fisher’s exact test *p* < 0.001).

Surgery was required in 21.5% (58/270) of the MET cohort and 50.4% (122/242) of the analgesic-only cohort (RD −28.9 pp, 95% CI −36.6 to −20.7; RR 0.43, 95% CI 0.33–0.55; OR 0.27, 95% CI 0.18–0.40). These figures mirror the primary outcome and are not independent evidence. Because early surgery precludes later spontaneous passage, the result reflects the overall management strategy, including the decision to operate, and not the drug alone.

### 3.4. Stone Size, Hydronephrosis and Spontaneous Passage

Spontaneous passage decreased with increasing stone-size category in both cohorts (Figure 2B, Table 3): 89.7%, 79.7% and 58.2% in the MET cohort and 77.3%, 44.0% and 21.6% in the analgesic-only cohort for stones of 4–5, >5–7 and >7–10 mm, respectively (association with size category: MET χ^2^(2) = 19.99, *p* < 0.001; analgesic-only χ^2^(2) = 40.58, *p* < 0.001; Cochran–Armitage trend test: Z = −4.32 and −6.31, both *p* < 0.001). This trend refers to size categories, not a per-millimeter gradient.

Within each size category, passage was higher in the MET cohort (Table 3). The absolute difference was 12.3 pp (95% CI 0.9–24.0) for 4–5 mm stones, 35.7 pp (95% CI 23.8–46.3) for >5–7 mm stones and 36.6 pp (95% CI 18.1–51.7) for >7–10 mm stones. For 4–5 mm stones, the conclusion depended on the test used (Pearson *p* = 0.033; Fisher’s exact *p* = 0.052; continuity-corrected *p* = 0.055) and should therefore be regarded as borderline. After Holm correction for the three size-specific comparisons, the Pearson *p*-values were 0.033, <0.001 and <0.001; the value for 4–5 mm is unchanged because it is the largest of the three ordered raw *p*-values. The numerically larger absolute and relative differences in larger stones did not provide evidence of effect modification on the odds-ratio scale (treatment × stone size interaction in the model with treatment and stone size, without hydronephrosis: likelihood-ratio test [LRT] χ^2^(2) = 1.74, *p* = 0.420).

Moderate hydronephrosis became more frequent as stones got larger in both cohorts (MET: 9.2%, 28.9% and 60.0%; χ^2^(2) = 42.34, *p* < 0.001; Cramér’s V = 0.396; analgesic-only: 9.3%, 28.4% and 60.8%; χ^2^(2) = 38.85, *p* < 0.001; V = 0.401). It was also linked to lower passage than absent or mild hydronephrosis: 28.2% (20/71) versus 58.5% (100/171) in the analgesic-only cohort (OR 0.28, 95% CI 0.15–0.51; RR 0.48, 95% CI 0.33–0.71) and 62.8% (49/78) versus 84.9% (163/192) in the MET cohort (OR 0.30, 95% CI 0.16–0.55; RR 0.74, 95% CI 0.62–0.89; both *p* < 0.001). Because the two factors are correlated, these crude figures partly reflect stone size, so the adjusted analysis below is the better guide.

### 3.5. Adjusted Analysis

**Figure 3:**
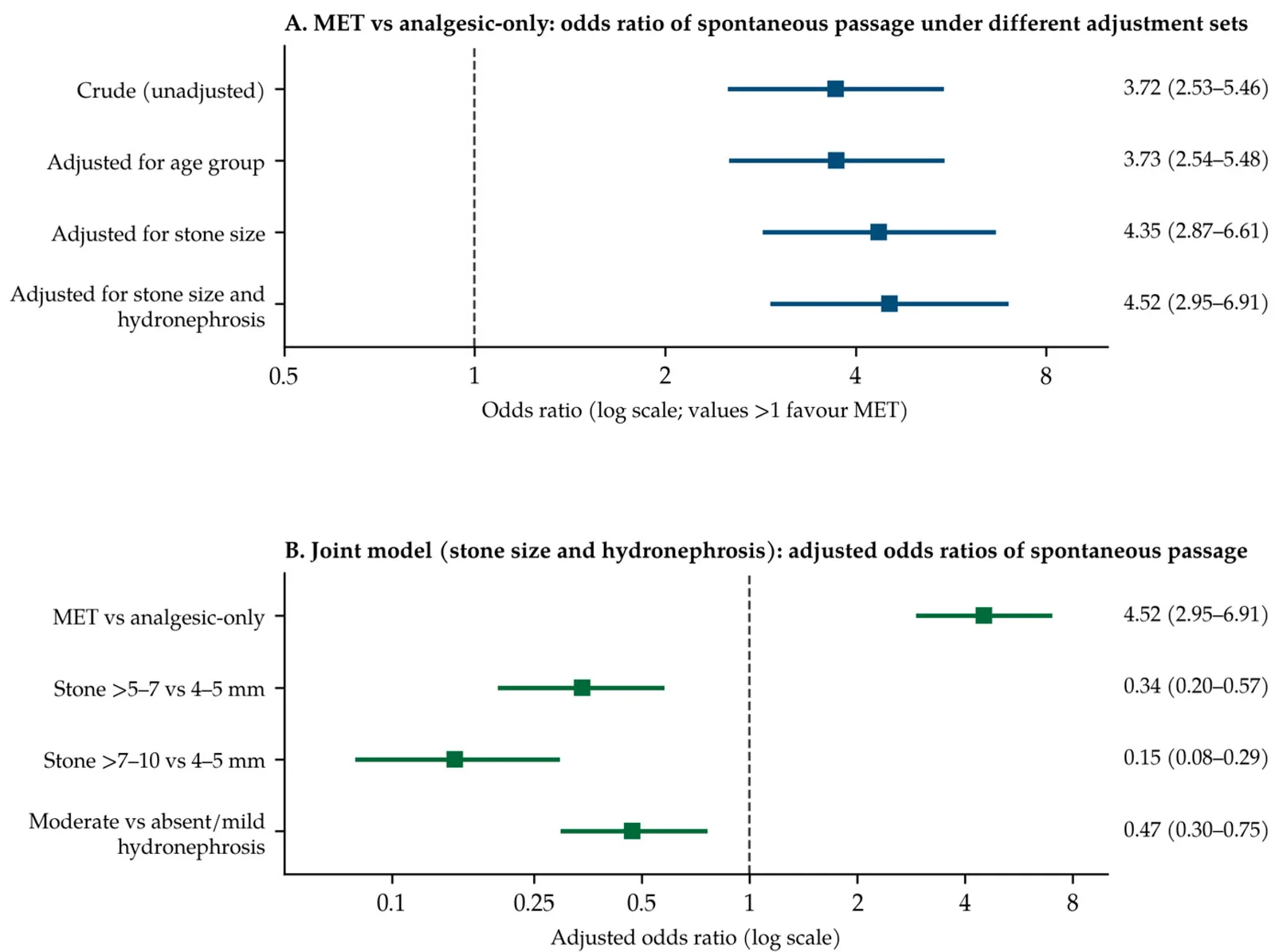
Adjusted odds ratios of spontaneous stone passage. (**A**) MET versus analgesic-only (reference) under different adjustment sets; each adjusted estimate comes from a separate model. (**B**) Mutually adjusted odds ratios in the joint model of treatment, stone size and hydronephrosis (*n* = 512). Lines show Wald 95% CIs on a logarithmic scale; the dashed line marks an odds ratio of 1.

To account for baseline differences between the cohorts, we fitted logistic regression models for spontaneous passage (Table 4, Figure 3). The available data were limited to the overall age distribution and the cross-tabulation of treatment, stone size and hydronephrosis, so age and the size–hydronephrosis variables had to be examined in separate models and not together in a single one. In the model adjusted for stone size and hydronephrosis, MET remained associated with higher odds of spontaneous passage (adjusted OR [aOR] 4.52, 95% CI 2.95–6.91; *p* < 0.001). Larger stones and moderate hydronephrosis were each linked to lower odds of passage. Adjusting for stone size alone (aOR 4.35, 95% CI 2.87–6.61) or for age group alone (aOR 3.73, 95% CI 2.54–5.48) gave estimates in the same range as the crude OR of 3.72. Age group itself was not associated with passage (11–14 years: aOR 1.12, 95% CI 0.63–2.02; 15–18 years: aOR 1.16, 95% CI 0.66–2.04; both compared with 5–10 years).

We found no sign of poor fit in the joint model (deviance 3.52 on 7 degrees of freedom, *p* = 0.833), although this test has limited power, and there were no empty cells, separation or convergence problems. These estimates are odds ratios, not risk ratios. Because treatment coincides completely with the calendar period (2016–2023 versus 2009–2015), statistical adjustment cannot separate the effect of the drug from the effect of the period, and unmeasured differences in imaging, surgical thresholds or follow-up practice may remain. The adjusted results should therefore be read as associations.

### 3.6. Age Groups and Exploratory Interaction Analyses

The age comparisons are exploratory. Observed passage was higher with MET than with analgesics alone in all three age groups (Table 5, Figure 2C). In the youngest group, the difference was not statistically significant (Pearson *p* = 0.097; Fisher’s exact *p* = 0.151, as in the original submission), but the wide CI is compatible with anything from no effect to a substantial benefit. Finding a significant result in one age group and not in another does not mean the effect differs between them, and the treatment × age interaction was not significant (LRT χ^2^(2) = 1.88, *p* = 0.390). The data therefore do not show that MET works better in older children, nor do they show that it works equally well at all ages, because the youngest group (*n* = 76) is too small to estimate the effect precisely.

**Figure 4:**
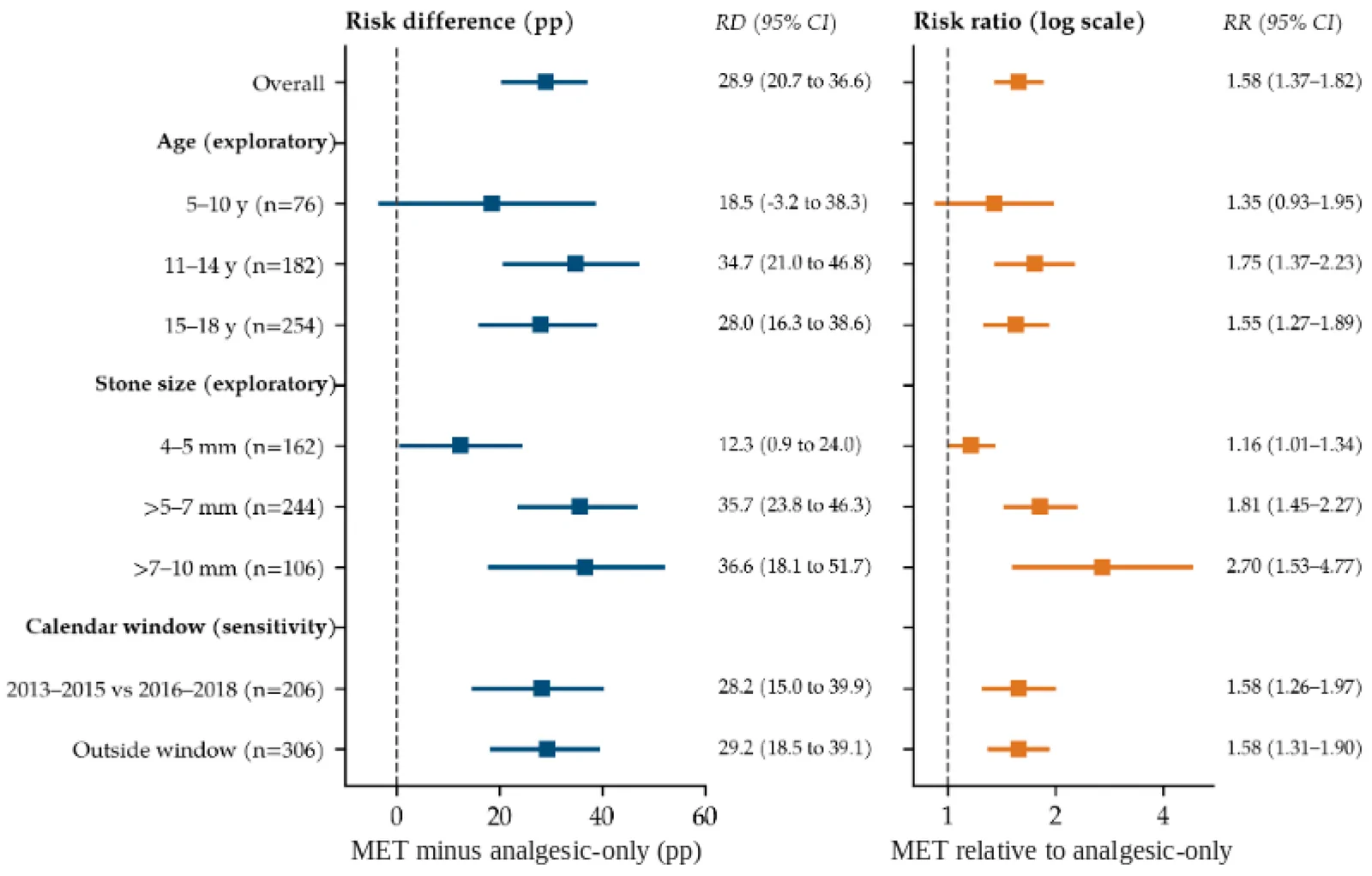
Unadjusted effect estimates for MET versus analgesic-only treatment by age group, stone-size category and calendar window: risk differences in percentage points (**left**) and risk ratios on a logarithmic scale (**right**) with 95% CIs. Subgroup and window analyses are exploratory. No interaction on the odds-ratio scale was detected by age (*p* = 0.390; model with treatment and age group) or stone size (*p* = 0.420; model with treatment and stone size).

In the joint model (treatment, stone size and hydronephrosis), we found no interaction between treatment and stone size (LRT χ^2^(2) = 1.92, *p* = 0.382, in the model that also includes hydronephrosis; the corresponding test without hydronephrosis gave *p* = 0.420), between treatment and hydronephrosis (χ^2^(1) = 0.03, *p* = 0.861), or between stone size and hydronephrosis (χ^2^(2) = 0.98, *p* = 0.613). Taken together, all pairwise interactions (χ^2^(5) = 2.95, *p* = 0.708) and the three-way interaction (χ^2^(2) = 0.57, *p* = 0.753) were also non-significant. These tests have limited power because some groups were small, so a non-significant result does not rule out an interaction. These tests assess heterogeneity on the odds-ratio scale and do not establish homogeneity of risk differences or risk ratios. Passage was numerically higher with MET in all six stone size and hydronephrosis groups (Supplementary Table S1), although the estimates are imprecise in the smallest groups. Figure 4 shows the unadjusted effect estimates across the subgroups. The subgroup *p*-values are not adjusted for multiplicity and should be read with that in mind, except for the Holm adjustment of the three stone-size tests described above.

### 3.7. Sensitivity Analysis by Calendar Window

To check that long-term changes in practice were not driving the result, we repeated the primary comparison in the three years just before (2013–2015, *n* = 107) and just after (2016–2018, *n* = 99) MET was introduced (Table 6). Spontaneous passage occurred in 76 of 99 patients (76.8%) with MET versus 52 of 107 (48.6%) with analgesics alone (RD 28.2 pp, 95% CI 15.0–39.9; RR 1.58, 95% CI 1.26–1.97; OR 3.49, 95% CI 1.92–6.38; Pearson χ^2^(1) = 17.34, *p* < 0.001), almost the same as in the whole cohorts. The remaining patients (MET 2019–2023, *n* = 171; analgesic-only 2009–2012, *n* = 135) showed a similar difference (RD 29.2 pp, 95% CI 18.5–39.1; RR 1.58, 95% CI 1.31–1.90), and there was no sign that the treatment effect differed between the two windows (LRT χ^2^(1) = 0.05, *p* = 0.820).

### 3.8. Timing of Surgery

Among the 180 patients in whom surgery was decided (58 in the MET cohort, 122 analgesic-only), the week of follow-up in which the decision was made was as follows (Table 7; χ^2^(2) = 4.05, *p* = 0.132).

This is only a descriptive comparison within a group defined by an outcome that occurs after treatment, so a non-significant result does not show that timing was the same, or that MET did not delay surgery. Counted against all included patients, a decision to operate in weeks 1–2 was made in 27 of 270 MET patients (10.0%) and 39 of 242 analgesic-only patients (16.1%), and in week 3, in 31 of 270 (11.5%) and 83 of 242 (34.3%).

### 3.9. Safety

Adverse effects of tamsulosin were not a prespecified outcome in the study protocol and were not systematically recorded. Parents were asked about possible adverse effects at follow-up visits, and no drug-related adverse effects were reported by parents in the MET cohort, the coverage of this informal reporting is unknown. Events during the treatment pathway, such as febrile infection, worsening obstruction, unplanned admission or urgent drainage, were not systematically recorded, so their rates cannot be estimated, and the absence of reports should not be read as a verified zero-event rate among 270 patients. Because no comparable information is available for the analgesic-only cohort, no comparative safety analysis was performed. The absence of reported adverse effects does not establish the safety of tamsulosin in this population.

## 4. Discussion

Despite the growing number of publications on MET in the pediatric population, consensus on recommendations remains limited due to heterogeneity among studies in methodology, choice of medication, treatment duration, dosage, patient age, stone size, and outcome assessment [5,6,19,20,21,22,23]. In the present prospective observational cohort study with a historical comparator, tamsulosin-based MET was associated with a higher observed rate of spontaneous passage of distal ureteral stones measuring 4–10 mm and, consequently, with a lower proportion of patients requiring ureteroscopy and lithotripsy in children and adolescents. Overall, spontaneous stone passage was achieved in 78.5% of patients in the MET group and 49.6% in the analgesic-treatment group (absolute difference 28.9 percentage points, 95% CI 20.7–36.6; risk ratio 1.58, 95% CI 1.37–1.82), whereas endoscopic treatment was required in 21.5% of patients in the MET group and 50.4% in the control group. Because every analyzed patient either passed the stone or underwent surgery, these two findings are complementary descriptions of the same outcome and not independent confirmations. The association remained after adjustment for stone size and hydronephrosis (adjusted odds ratio 4.52, 95% CI 2.95–6.91) and in an analysis restricted to the three years before and after the introduction of MET (risk ratio 1.58, 95% CI 1.26–1.97). However, because treatment and calendar period coincide, these findings should be read as associations and not as an isolated pharmacological effect of tamsulosin.

The available literature on MET in the pediatric population showed that our results are consistent with previously published findings. Early studies reported improved spontaneous stone passage in patients receiving tamsulosin. Mokhless et al. demonstrated the efficacy of tamsulosin in a prospective randomized pediatric study, while Tasian et al. reported increased spontaneous passage in a multi-institutional pediatric cohort treated with tamsulosin compared with analgesics alone [17,18]. Subsequently published systematic reviews and meta-analyses confirmed that alpha-blockers facilitate stone expulsion in children with distal ureteral stones and shorten the time to expulsion [19,20,21,22,24]. The presented cohort study contributes to the existing literature by providing single-center, real-world evidence from routine clinical practice supporting the concept that, in selected patients with distal ureteral stones, MET may play a therapeutic role. It does not, however, provide new evidence of efficacy beyond that of the randomized trials and meta-analyses cited above.

The clinical significance of these findings is primarily reflected in the lower observed need for surgical treatment in the pediatric population, which, although effective, requires anesthesia and endoscopic instrumentation of the urinary tract, thereby exposing patients to the potential risks and complications associated with these interventions [5,6,22]. Therefore, any treatment with an acceptable safety profile that can prevent or reduce the need for surgery is of considerable importance in the developing urinary tract. In this study, approximately one in five patients treated with MET required ureteroscopy with lithotripsy, whereas in the analgesic-treatment group, almost every second patient underwent the procedure. Because early surgery precludes later spontaneous passage, the observed outcome reflects the combined management strategy, including the decision to operate, and not the effect of the drug alone. This reduction in the need for operative intervention is particularly relevant given that the inclusion criteria comprised stones measuring 4–10 mm, as it represents the subgroup in which the decision between non-operative and operative management is most uncertain. Stones measuring up to 4 mm and located in the distal ureter have a high likelihood of spontaneous passage, making the measurable effect of MET difficult to demonstrate [5,7,8]. At the other end of the spectrum, stones exceeding 10 mm are less likely to pass spontaneously and are associated with more frequent emergency department visits due to worsening symptoms and progressive obstruction, thereby increasing the importance of endoscopic treatment in such cases [5,7,8]. Therefore, distal ureteral stones measuring between 4 and 10 mm represent a clinical “gray zone” in treatment protocols, in which spontaneous expulsion cannot be assumed, but surgical intervention may still be avoided. Accordingly, most studies evaluating the effects of MET have focused on this subgroup of patients [17,18,19,20,21,22].

Stone size was the strongest measured determinant of spontaneous passage. In both cohorts, passage decreased across the size categories (MET 89.7%, 79.7% and 58.2%; analgesic-only 77.3%, 44.0% and 21.6% for stones of 4–5, >5–7 and >7–10 mm), in line with the pediatric literature [7,8]. The difference between cohorts was smaller for 4–5 mm stones (12.3 percentage points, 95% CI 0.9–24.0; borderline significance depending on the test used) than for >5–7 and >7–10 mm stones (35.7 and 36.6 percentage points) (Table 3). This pattern is in the same direction as a meta-analysis of adult trials, in which the benefit of alpha-blockers was confined to larger stones (risk ratio 1.57, 95% CI 1.17–2.27), with no clear benefit for smaller stones (risk ratio 1.19, 95% CI 1.00–1.48) [10]. However, we found no statistical evidence that the effect differed by stone size (interaction *p* = 0.420 in the model with treatment and stone size, on the odds-ratio scale), and stone size could be linked to the outcome only in categories, so this observation remains exploratory. Moderate hydronephrosis, which became more frequent with increasing stone size, was associated with lower passage in both cohorts and also after adjustment for stone size (adjusted odds ratio 0.47, 95% CI 0.30–0.75). The pediatric literature on hydronephrosis is conflicting: it was associated with lower passage in one series [7] but not in another [8] and differences in definitions, imaging and sample size may account for this.

Observed passage was higher with MET in all age groups examined in this study, however, statistically significant differences were observed only in the 11–14- and 15–18-year age groups (Table 5). A significant result in one subgroup and a non-significant result in another does not demonstrate that the treatment effect differs between age groups, and no treatment-by-age interaction was found (*p* = 0.390). The absence of a statistically significant difference in the youngest age group should be interpreted with caution. It does not necessarily indicate a lack of pharmacological benefit but may instead reflect the smaller number of patients. Other possible explanations, such as the smaller average ureteral diameter and consequently larger stone-to-ureter ratio, lower tolerance of symptoms, and a greater clinical tendency to proceed with invasive treatment to prevent the consequences of obstruction in the developing urinary tract and to relieve patient discomfort, remain hypotheses, as they were not measured in this cohort. Available meta-analyses have not established an age-dependent response to the applied therapy [21,22]. Taken together, these findings suggest that the youngest subgroup should not be regarded as unsuitable for an initially conservative treatment approach.

Determining when to proceed with invasive procedures is perhaps the most important practical clinical question in the management of distal ureteral stones in children. Most studies evaluating the efficacy of MET have assessed treatment success within a period of 3 to 4 weeks, while protocol-based discussions have consistently relied on the recommendation that the decision to pursue conservative treatment should be individualized, taking into account stone size, symptom control and tolerance, the likelihood of spontaneous passage, and the risk of infection and consequences of progressive obstruction [5,6,22,25]. In our study, a clear follow-up protocol was established for patients diagnosed with distal ureteral calculi. Patients underwent scheduled ultrasound examinations on days 7, 14, and 21, with the possibility of additional unscheduled assessments if symptoms worsened despite the applied treatment. The same protocol and the same criteria for surgery (worsening of symptoms, impaired renal function, signs of infection, or pain that could not be controlled with analgesics) were applied in both cohorts. Protocolized ultrasound follow-up of patients with distal ureteral stones is an important step in monitoring and evaluating the success of conservative treatment, as symptom resolution and patient-reported stone passage do not reliably confirm stone expulsion [26]. Among patients who required surgical intervention despite conservative therapy, the distribution of the week of follow-up in which surgery was decided did not differ significantly between the groups (*p* = 0.132). This comparison is purely descriptive and limited to patients who ended up needing surgery. A non-significant difference does not prove that timing was the same, nor that MET did not delay operative treatment. Likewise, when the need for surgical intervention arises, it should not be considered a failure of MET, but only one of the outcomes of the overall treatment of distal ureteral stones. The present study describes the performance of an institutional 21-day pathway, it did not compare alternative durations and does not show that three weeks is optimal or that it prevents renal damage.

As in other published studies, no drug-related adverse effects were reported by parents in the present cohort [17,18]. However, adverse effects were not systematically recorded, comparable information is not available for the historical cohort, and this finding does not establish the safety of tamsulosin in this population. Although uncommon, meta-analyses have reported minor adverse effects associated with alpha-adrenergic blockers, including headache, dizziness, nasal congestion, somnolence, and gastrointestinal symptoms. In contrast, serious adverse events are exceedingly rare [21,22,24]. In comparative studies evaluating different alpha-blockers, the use of silodosin has been associated with a higher rate of the primary outcome and a shorter time to expulsion of stones located in the distal third of the ureter [27]. Taking this into consideration, future studies should focus not only on the efficacy of this drug class, but also on identifying which agent, dose, and treatment duration provide the most favorable balance between efficacy and safety.

The present study has several limitations. Although data on patients receiving MET were collected prospectively, the comparison group was a historical cohort treated with analgesics alone, so there was no randomization. Treatment and calendar period coincide completely: every MET patient was treated in 2016 or later and every control patient before that. Although the hospital protocol did not change apart from the introduction of tamsulosin therapy, over such a long comparison period, we cannot exclude the influence of time, advances in imaging methods, referral patterns, surgical thresholds and individual physician decision-making, parental support and persistence with treatment, and the reliability of reported data. Neither adjustment for measured characteristics nor the analysis restricted to the years around the introduction of MET can separate the effect of the drug from the effect of the period.

Our data are also limited in other ways. Age (in years) and stone size (in whole millimeters, measured by ultrasonography) were available as separate distributions in each cohort but not cross-classified with each other or with the outcome, so the outcome analyses used age and stone-size categories. Body weight was not available for this analysis, laterality, renal function and symptom duration were not recorded and the date and reason for surgery were not extracted. Apart from age, sex, stone size and hydronephrosis, no other baseline characteristics could be compared between the cohorts. The adjusted analysis was therefore restricted to separate models for age and for stone size with hydronephrosis, none of which adjust simultaneously for all three, and residual confounding is likely. All children received the same dose of tamsulosin, so we could not examine dose–response relationships. Adherence to tamsulosin was neither recorded nor measured. Confirmation of stone passage relied on ultrasonography, which has limited sensitivity for detecting urinary stones in children [28], so failure to visualize a stone may overestimate passage in both cohorts. Equivocal ultrasound findings were handled by repeat examination according to institutional protocol, but their number was not separately documented, so differential ascertainment of passage between the two periods cannot be excluded. The study was conducted in a single center, no formal sample size calculation was performed, and the number of patients screened was not recorded. In addition, 57 of 569 patients (10.0%; 8.5% in the MET and 11.7% in the analgesic-only cohort) were lost to follow-up and excluded, so the reported proportions apply to patients with documented outcomes, and attrition bias cannot be excluded. The comparisons by age group, by stone size (apart from the Holm adjustment of the three stone-size tests) and in the six supplementary strata were exploratory and not otherwise adjusted for multiplicity, and the youngest age group was small. The actual indications for surgery in individual patients were not extracted, so their comparability between the two periods cannot be verified from the available dataset. Because the inclusion criteria covered a defined range of stone size, our results cannot be extended to stones outside that range, nor to stones of the same size in the proximal or mid-ureter. Finally, safety was not a prespecified outcome in the study protocol, and the study was not designed to assess it. Adverse effects were obtained only by asking parents, without systematic recording, and the absence of reported adverse effects, although encouraging and consistent with previous studies, does not establish the safety of tamsulosin.

## 5. Conclusions

Tamsulosin-based MET was associated with a higher observed rate of spontaneous passage of distal ureteral stones and a lower proportion of patients undergoing ureteroscopy with lithotripsy compared with historical analgesic therapy alone in children and adolescents with distal ureteral stones measuring 4–10 mm. These findings require cautious interpretation because of the non-contemporaneous comparison and potential confounding by calendar period, stone characteristics and intervention practices. Observed passage was higher with MET in all three age groups, with the greatest uncertainty in the youngest children, and no age-dependent difference in effect was demonstrated. This single-center, real-world experience supports considering tamsulosin as part of conservative treatment for selected children with distal ureteral stones. Confirmation in contemporaneous or randomized comparisons is still needed.

## Figures and Tables

**Table 1:** Baseline characteristics of the prospective MET cohort and the historical analgesic-only cohort.

Characteristic	MET	Analgesic-Only	*p*
***Whole cohorts (MET 2016–2023, N = 270; analgesic-only 2009–2015, N = 242)***			
Age, years, median (IQR)	14 (12–17)	15 (12–17)	0.907
Stone size, mm, median (IQR)	6 (5–7)	6 (5–7)	0.802
Age group, years			0.880
5–10	42 (15.6)	34 (14.0)	
11–14	96 (35.6)	86 (35.5)	
15–18	132 (48.9)	122 (50.4)	
Female sex	122 (45.2)	105 (43.4)	0.683
Stone size category			0.952
4–5 mm	87 (32.2)	75 (31.0)	
>5–7 mm	128 (47.4)	116 (47.9)	
>7–10 mm	55 (20.4)	51 (21.1)	
Moderate hydronephrosis	78 (28.9)	71 (29.3)	0.911
***Adjacent three-year windows (MET 2016–2018, N = 99; analgesic-only 2013–2015, N = 107)***			
Age group, years			0.969
5–10	15 (15.2)	15 (14.0)	
11–14	35 (35.4)	39 (36.4)	
15–18	49 (49.5)	53 (49.5)	
Female sex	44 (44.4)	46 (43.0)	0.834
Stone size category			0.990
4–5 mm	30 (30.3)	32 (29.9)	
>5–7 mm	49 (49.5)	54 (50.5)	
>7–10 mm	20 (20.2)	21 (19.6)	
Moderate hydronephrosis	30 (30.3)	26 (24.3)	0.333

Values are *n* (%) unless stated otherwise. p-values: Pearson χ^2^ test without continuity correction; for age group and stone size, the test refers to the whole distribution (2 degrees of freedom); for sex and hydronephrosis, the displayed category is compared with its complement. Age and stone size (in whole years and millimeters) are given as median (IQR) and compared with the Mann–Whitney U test. Hydronephrosis was graded with the Society for Fetal Urology (SFU) system and recorded as absent/mild (grades 0–1) versus moderate (grades 2–3). A non-significant p-value is not evidence of equivalence. MET, medical expulsive therapy.

**Table 2:** Primary outcome and surgery (decision to operate by day 21) in the MET and analgesic-only cohorts.

Outcome	MET (*N* = 270)	Analgesic-Only (*N* = 242)	RD, pp (95% CI)	RR (95% CI)	OR (95% CI)	*p*
Spontaneous passage	212 (78.5%; 73.2–83.0)	120 (49.6%; 43.3–55.8)	28.9 (20.7 to 36.6)	1.58 (1.37–1.82)	3.72 (2.53–5.46)	<0.001
Surgery (decision to proceed to ureteroscopy with laser lithotripsy by day 21)	58 (21.5%; 17.0–26.8)	122 (50.4%; 44.2–56.7)	−28.9 (−36.6 to −20.7)	0.43 (0.33–0.55)	0.27 (0.18–0.40)	<0.001

Values are *n* (%; 95% Wilson CI). RD, risk difference (MET minus analgesic-only) in percentage points, Newcombe CI; RR, risk ratio, Katz log CI; OR, odds ratio, Wald log CI; the analgesic-only cohort is the reference. *p*: Pearson χ^2^ test without continuity correction (Fisher’s exact test: *p* < 0.001). Passage and surgery are complementary outcomes and are not independent tests of efficacy.

**Table 3:** Spontaneous passage during the 21-day period by stone-size category (exploratory subgroup comparison).

Stone Size	MET, *n*/*N* (%)	Analgesic-Only, *n*/*N* (%)	RD, pp (95% CI)	RR (95% CI)	OR (95% CI)	p Pearson	p Fisher
All sizes	212/270 (78.5)	120/242 (49.6)	28.9 (20.7 to 36.6)	1.58 (1.37–1.82)	3.72 (2.53–5.46)	<0.001	<0.001
4–5 mm	78/87 (89.7)	58/75 (77.3)	12.3 (0.9 to 24.0)	1.16 (1.01–1.34)	2.54 (1.06–6.10)	0.033	0.052
>5–7 mm	102/128 (79.7)	51/116 (44.0)	35.7 (23.8 to 46.3)	1.81 (1.45–2.27)	5.00 (2.84–8.80)	<0.001	<0.001
>7–10 mm	32/55 (58.2)	11/51 (21.6)	36.6 (18.1 to 51.7)	2.70 (1.53–4.77)	5.06 (2.15–11.91)	<0.001	<0.001

RD, RR and OR: MET versus analgesic-only (reference); CI methods as in Table 2. Stone-size subgroup analyses are exploratory; Holm adjustment was applied to the three Pearson tests (adjusted *p* = 0.033, <0.001 and <0.001; the value for 4–5 mm equals the raw value because it is the largest of the three ordered *p*-values). The 95% CIs are not obtained by inversion of Fisher’s exact test, so a CI excluding no effect may coexist with a Fisher *p* slightly above 0.05.

**Table 4:** Logistic regression models for spontaneous stone passage (adjusted odds ratios).

Model	Predictor	aOR (95% CI)	*p*
***Model 1: Adjusted for age group (n = 512)***			
	MET vs. analgesic-only	3.73 (2.54–5.48)	<0.001
	Age 11–14 vs. 5–10 years	1.12 (0.63–2.02)	0.698
	Age 15–18 vs. 5–10 years	1.16 (0.66–2.04)	0.595
***Model 2: Adjusted for stone size (n = 512)***			
	MET vs. analgesic-only	4.35 (2.87–6.61)	<0.001
	Stone >5–7 vs. 4–5 mm	0.29 (0.17–0.49)	<0.001
	Stone >7–10 vs. 4–5 mm	0.11 (0.06–0.20)	<0.001
***Model 3: Adjusted for stone size and hydronephrosis (n = 512)***			
	MET vs. analgesic-only	4.52 (2.95–6.91)	<0.001
	Stone >5–7 vs. 4–5 mm	0.34 (0.20–0.57)	<0.001
	Stone >7–10 vs. 4–5 mm	0.15 (0.08–0.29)	<0.001
	Moderate vs. absent/mild hydronephrosis	0.47 (0.30–0.75)	0.001

aOR, adjusted odds ratio of spontaneous passage (Wald 95% CI; maximum-likelihood logistic regression on the available cross-classified frequencies). Reference categories: analgesic-only, age 5–10 years, stone 4–5 mm, absent/mild hydronephrosis. Each model was fitted separately; the models are not mutually adjusted for age. Model 3 fit: deviance 3.52, 7 df, *p* = 0.833. Odds ratios are not risk ratios.

**Table 5:** Spontaneous passage during the 21-day period by age group (exploratory subgroup comparison).

Age Group	MET, *n*/*N* (%)	Analgesic-Only, *n*/*N* (%)	RD, pp (95% CI)	RR (95% CI)	OR (95% CI)	p Pearson	p Fisher
5–10 years	30/42 (71.4)	18/34 (52.9)	18.5 (−3.2 to 38.3)	1.35 (0.93–1.95)	2.22 (0.86–5.74)	0.097	0.151
11–14 years	78/96 (81.3)	40/86 (46.5)	34.7 (21.0 to 46.8)	1.75 (1.37–2.23)	4.98 (2.56–9.69)	<0.001	<0.001
15–18 years	104/132 (78.8)	62/122 (50.8)	28.0 (16.3 to 38.6)	1.55 (1.27–1.89)	3.59 (2.08–6.22)	<0.001	<0.001

RD, RR and OR: MET versus analgesic-only (reference); CI methods as in Table 2. Age-group analyses are exploratory and unadjusted for multiplicity. Treatment × age interaction (model with treatment and age group, odds-ratio scale): LRT χ^2^(2) = 1.88, *p* = 0.390. The difference between the Pearson and Fisher *p*-values in the youngest group arises from the test and not from different frequencies.

**Table 6:** Sensitivity analysis of spontaneous passage by calendar window.

Sample	MET, *n*/*N* (%)	Analgesic-Only, *n*/*N* (%)	RD, pp (95% CI)	RR (95% CI)	OR (95% CI)	*p*
All patients (MET 2016–2023 vs. analgesic-only 2009–2015)	212/270 (78.5)	120/242 (49.6)	28.9 (20.7 to 36.6)	1.58 (1.37–1.82)	3.72 (2.53–5.46)	<0.001
Adjacent windows (MET 2016–2018 vs. analgesic-only 2013–2015)	76/99 (76.8)	52/107 (48.6)	28.2 (15.0 to 39.9)	1.58 (1.26–1.97)	3.49 (1.92–6.38)	<0.001
Remaining patients (MET 2019–2023 vs. analgesic-only 2009–2012)	136/171 (79.5)	68/135 (50.4)	29.2 (18.5 to 39.1)	1.58 (1.31–1.90)	3.83 (2.32–6.32)	<0.001

CI methods as in Table 2; *p*: Pearson χ^2^ test. The remaining patients were obtained by subtracting the adjacent windows from the whole cohorts. The whole-cohort row contains the adjacent windows and is not an independent sample.

**Table 7:** Week of follow-up in which the decision to operate was made among patients in whom surgery was decided (surgery performed in the following days), overall and by age group.

Age Group	MET wk 1	MET wk 2	MET wk 3	MET Total	Analg. wk 1	Analg. wk 2	Analg. wk 3	Analg. Total
All operated	9 (15.5)	18 (31.0)	31 (53.4)	58	16 (13.1)	23 (18.9)	83 (68.0)	122
5–10 years	2 (16.7)	8 (66.7)	2 (16.7)	12	4 (25.0)	5 (31.3)	7 (43.8)	16
11–14 years	3 (16.7)	4 (22.2)	11 (61.1)	18	5 (10.9)	5 (10.9)	36 (78.3)	46
15–18 years	4 (14.3)	6 (21.4)	18 (64.3)	28	7 (11.7)	13 (21.7)	40 (66.7)	60

Values are n (% of patients in the row in whom surgery was decided). Overall distribution compared with Pearson χ^2^(2) = 4.05, *p* = 0.132 (descriptive only). Analg., analgesic-only cohort.

## Data Availability

The minimal dataset supporting the main findings and statistical analyses of this study is provided as Supplementary Dataset S2. The dataset contains aggregated, de-identified study data. Individual-level patient data are not publicly available due to ethical and privacy considerations related to pediatric clinical data. The source hospital records remain available for verification upon reasonable request.

## Supplementary Materials

The following supporting information can be downloaded at: https://www.mdpi.com/article/10.3390/healthcare14213632/s1, Supplementary Table S1—Spontaneous passage during the 21-day pathway in the six strata of stone size and hydronephrosis. Supplementary Dataset S2—Aggregated, de-identified frequencies used for all analyses (Excel), STROBE checklist.

## Abbreviations

The following abbreviations are used in this manuscript:

aOR	adjusted odds ratio
CI	confidence interval
LRT	likelihood-ratio test
MET	medical expulsive therapy
OR	odds ratio
pp	percentage points
RD	risk difference
RR	risk ratio
SFU	Society for Fetal Urology
STROBE	Strengthening the Reporting of Observational Studies in Epidemiology
