# Supplementary material for: A Tale of Two Pathways: Tamsulosin MET Versus Historical Analgesic-Only Therapy for Pediatric Distal Ureteral Stones
Source: Healthcare (Basel). 2026 Oct 26;14(21):3632. doi: 10.3390/healthcare14213632 (PMC13762601; doi:10.3390/healthcare14213632)
Supplement: Supplementary file 1 [file healthcare-14-03632-s001.zip › Supplementary Table S1.pdf]

Supplementary Materials

A Tale of Two Pathways: Tamsulosin MET versus Historical Analgesic-only therapy for Pediatric distal ureteral stones

Table S1. Spontaneous passage during the 21-day pathway in the six strata of stone size and hydronephrosis (exploratory).

Stone size	Hydro-nephrosis	MET, n/N (%)	Analgesic-only, n/N (%)	RD, pp (95% CI)	RR (95% CI)	OR (95% CI)	p Fisher
4–5 mm	Absent/mild	72/79 (91.1)	53/68 (77.9)	13.2 (1.5 to 25.3)	1.17 (1.01–1.35)	2.91 (1.11–7.64)	0.036
4–5 mm	Moderate	6/8 (75.0)	5/7 (71.4)	3.6 (−36.1 to 43.3)	1.05 (0.57–1.94)	1.20 (0.12–11.87)	1.000
>5–7 mm	Absent/mild	75/91 (82.4)	40/83 (48.2)	34.2 (20.3 to 46.5)	1.71 (1.34–2.18)	5.04 (2.53–10.05)	<0.001
>5–7 mm	Moderate	27/37 (73.0)	11/33 (33.3)	39.6 (16.3 to 57.5)	2.19 (1.30–3.69)	5.40 (1.94–15.05)	0.002
>7–10 mm	Absent/mild	16/22 (72.7)	7/20 (35.0)	37.7 (7.6 to 59.7)	2.08 (1.09–3.98)	4.95 (1.33–18.41)	0.029
>7–10 mm	Moderate	16/33 (48.5)	4/31 (12.9)	35.6 (13.0 to 53.6)	3.76 (1.41–10.01)	6.35 (1.82–22.23)	0.003

MET, medical expulsive therapy; RD, risk difference (MET minus analgesic-only) in percentage points (pp), Newcombe 95% CI; RR, risk ratio, Katz log 95% CI; OR, odds ratio, Wald log 95% CI. The analgesic-only cohort is the reference. Exploratory analysis; *p*-values are not corrected for six comparisons. Differences between strata should be assessed with interaction tests (see Section 3.6) and not by comparing individual *p*-values. Intervals in the smallest groups are imprecise and descriptive.
