# Supplementary material for: A Tale of Two Pathways: Tamsulosin MET Versus Historical Analgesic-Only Therapy for Pediatric Distal Ureteral Stones
Source: Healthcare (Basel). 2026 Oct 26;14(21):3632. doi: 10.3390/healthcare14213632 (PMC13762601; doi:10.3390/healthcare14213632)
Supplement: Supplementary file 1 [file healthcare-14-03632-s001.zip › STROBE checklist.pdf]

STROBE Statement—Checklist of items that should be included in reports of cohort studies

A Tale of Two Pathways: Tamsulosin MET versus Historical Analgesic-only therapy for Pediatric distal ureteral stones

Page and line numbers refer to the revised manuscript with continuous line numbering.

Item No	Recommendation	Page and line numbers
Title and abstract		
1	(a) Indicate the study's design with a commonly used term in the title or the abstract	Title: p. 1, lines 2–3 (historical comparator). Abstract: p. 3, lines 28–29 ("single-center prospective observational cohort study with a historical comparator").
	(b) Provide in the abstract an informative and balanced summary of what was done and what was found	Abstract: pp. 3–4, lines 24–48.
Introduction		
2 Background/rationale	Explain the scientific background and rationale for the investigation being reported	Introduction: pp. 4–6, lines 53–108.
3 Objectives	State specific objectives, including any prespecified hypotheses	pp. 5–6, lines 102–108 (aim stated; no formal prespecified hypothesis).
Methods		
4 Study design	Present key elements of study design early in the paper	Methods, 2.1: p. 6, lines 111–123.
5 Setting	Describe the setting, locations, and relevant dates, including periods of recruitment, exposure, follow-up, and data collection	Setting, locations and periods of recruitment (2016–2023 and 2009–2015): p. 6, lines 111–118. Exposure: p. 7, lines 144–153. Follow-up: p. 7, lines 155–165.
6 Participants	(a) Give the eligibility criteria, and the sources and methods of selection of participants. Describe methods of follow-up	Eligibility and exclusion criteria: pp. 6–7, lines 125–142. Sources and selection (consecutive patients; hospital medical records): p. 6, lines 113–118. Follow-up: p. 7, lines 155–165.
	(b) For matched studies, give matching criteria and number of exposed and unexposed	Not applicable (no matching).
7 Variables	Clearly define all outcomes, exposures, predictors, potential confounders, and effect modifiers. Give diagnostic criteria, if applicable	Outcomes (primary: confirmed spontaneous passage during the 21-day conservative management pathway, before a decision to proceed to ureteroscopy; secondary: surgery = decision to proceed to ureteroscopy by day 21, and week of the decision; weeks defined as days 1–7, 8–14, 15–21): pp. 7–8, lines 166–176. Exposure (tamsulosin and analgesia): p. 7, lines 144–153. Predictors, confounders and effect modifiers (stone size, hydronephrosis, age group): p. 6, lines 128–129 and 135–138, p. 7, line 139, and p. 9, lines 202–212. Diagnostic criteria, distal ureter and hydronephrosis grading: pp. 6–7, lines 125–142.

Item No	Recommendation	Page and line numbers
8* Data sources/ measurement	For each variable of interest, give sources of data and details of methods of assessment (measurement). Describe comparability of assessment methods if there is more than one group	Sources: hospital patient records (prospective and retrospective), available for verification: p. 6, lines 116–118. Stone size and hydronephrosis measurement: p. 6, lines 128–129 and 135–137. Same follow-up protocol and criteria for surgery in both cohorts, ultrasound confirmation of passage and repeat examination for unclear findings: p. 7, lines 155–161 and pp. 7–8, lines 166–171. Verification that surgery was performed in all patients in whom it was decided: p. 7, lines 162–165 and p. 10, lines 246–248. Ascertainment limitation: p. 26, lines 577–582.
9 Bias	Describe any efforts to address potential sources of bias	Consecutive inclusion and identical eligibility criteria: p. 6, line 113 and pp. 6–7, lines 125–142. Unchanged protocol except tamsulosin: p. 6, lines 118–119. Adjusted analysis and calendar-window sensitivity analysis: p. 9, lines 202–206 and 214–216. Limitations: p. 26, lines 555–565 and pp. 26–27, lines 566–597.
10 Study size	Explain how the study size was arrived at	No formal sample size calculation; all consecutive eligible patients included: p. 6, lines 121–123; p. 26, lines 582–583.
11 Quantitative variables	Explain how quantitative variables were handled in the analyses. If applicable, describe which groupings were chosen and why	Age (years) and stone size (mm) are summarized as median (IQR): p. 11, lines 258–261 and Table 1, p. 12, line 265. Age groups (5–10, 11–14, 15–18 years; chosen for descriptive purposes): pp. 6–7, lines 138–139. Stone-size categories (4–5, >5–7, >7–10 mm) and hydronephrosis (absent/mild, SFU grades 0–1, vs. moderate, grades 2–3): p. 6, lines 128–129 and 135–137; Table 1, p. 12, line 265.
12 Statistical methods	(a) Describe all statistical methods, including those used to control for confounding	p. 9, lines 198–220 (tests, effect measures and confidence intervals; logistic regression: p. 9, lines 202–206).
	(b) Describe any methods used to examine subgroups and interactions	p. 9, lines 207–212 (interactions); p. 9, lines 216–218 (subgroups exploratory; Holm adjustment for stone size).
	(c) Explain how missing data were addressed	Patients whose outcome could not be determined (lost to follow-up) were excluded: p. 9, lines 213–214; p. 10, lines 237–241. All analysed patients had complete data for the analysed variables (Table 1, p. 12, line 265).
	(d) If applicable, explain how loss to follow-up was addressed	Patients lost to follow-up were excluded and the analysis restricted to patients with documented outcomes: p. 9, lines 213–214; Results, p. 10, lines 235–241; Figure 1, p. 11, lines 250–254; Limitations (possible attrition bias): pp. 26–27, lines 583–586.
	(e) Describe any sensitivity analyses	p. 9, lines 214–216 (calendar-window analysis).
Results		
13* Participants	(a) Report numbers of individuals at each stage of study—eg numbers potentially eligible, examined for eligibility, confirmed eligible, included in the study, completing follow-up, and analysed	Results, p. 10, lines 235–248; Figure 1, p. 11, lines 250–254 (numbers screened were not recorded: p. 10, line 242).

Item No	Recommendation	Page and line numbers
	(b) Give reasons for non-participation at each stage	Lost to follow-up: p. 10, lines 237–239 and Figure 1, p. 11, lines 250–254; exclusion criteria: p. 6, lines 130–138.
	(c) Consider use of a flow diagram	Figure 1, p. 11, lines 250–254.
14* Descriptive data	(a) Give characteristics of study participants (eg demographic, clinical, social) and information on exposures and potential confounders	Table 1, p. 12, line 265; text p. 11, lines 256–264.
	(b) Indicate number of participants with missing data for each variable of interest	Table 1, p. 12, line 265 (all variables complete for N = 270 and N = 242); variables not recorded: p. 7, lines 140–142; p. 26, lines 566–572.
	(c) Summarise follow-up time (eg, average and total amount)	Fixed observation window of 21 days with reassessment on days 7, 14 and 21: p. 7, lines 151–153 and 155–157; Figure 1, p. 11, lines 250–254.
15* Outcome data	Report numbers of outcome events or summary measures over time	Table 2, p. 14, line 287; text p. 13, lines 272–286; timing of surgery: Table 7, p. 21, line 408 and pp. 21–22, lines 405–416.
16 Main results	(a) Give unadjusted estimates and, if applicable, confounder-adjusted estimates and their precision (eg, 95% confidence interval). Make clear which confounders were adjusted for and why they were included	Unadjusted: Table 2, p. 14, line 287. Adjusted: p. 16, lines 329–340 and p. 17, lines 342–348; Table 4, p. 17, line 350; Figure 3, p. 18, line 355. Rationale for covariates: p. 16, lines 329–333.
	(b) Report category boundaries when continuous variables were categorized	Stone-size categories 4–5, >5–7, >7–10 mm and age groups: Table 1, p. 12, line 265; Table 3, p. 15, line 315; Table 5, p. 19, line 368.
	(c) If relevant, consider translating estimates of relative risk into absolute risk for a meaningful time period	Risk differences (percentage points) with 95% CI over the 21-day pathway: Table 2, p. 14, line 287; p. 13, lines 272–286.
17 Other analyses	Report other analyses done—eg analyses of subgroups and interactions, and sensitivity analyses	Stone size: Table 3, p. 15, line 315. Age: Table 5, p. 19, line 368. Interactions: p. 19, lines 372–385. Forest plot: Figure 4, p. 20, line 387. Calendar-window sensitivity: p. 20, lines 392–400 and Table 6, p. 21, line 401. Timing of surgery: Table 7, p. 21, line 408. Six strata: Supplementary Table S1.
Discussion		
18 Key results	Summarise key results with reference to study objectives	pp. 22–23, lines 429–447.
19 Limitations	Discuss limitations of the study, taking into account sources of potential bias or imprecision. Discuss both direction and magnitude of any potential bias	p. 26, lines 555–565 and pp. 26–27, lines 566–597 (historical comparator and period effects, residual confounding, missing baseline variables, loss to follow-up, exploratory subgroups, safety data).
20 Interpretation	Give a cautious overall interpretation of results considering objectives, limitations, multiplicity of analyses, results from similar studies, and other relevant evidence	Discussion: pp. 22–26, lines 429–554; Conclusions: p. 27, lines 599–609.
21 Generalisability	Discuss the generalisability (external validity) of the study results	p. 26, lines 582–583 and p. 27, lines 591–593 (single center; results not extendable to other stone sizes or locations); pp. 23–24, lines 471–483.
Other information		

Item No	Recommendation	Page and line numbers
22 Funding	Give the source of funding and the role of the funders for the present study and, if applicable, for the original study on which the present article is based	Funding statement: p. 28, line 621 (no external funding).

*Give information separately for exposed and unexposed groups.

Note: An Explanation and Elaboration article discusses each checklist item and gives methodological background and published examples of transparent reporting. The STROBE checklist is best used in conjunction with this article (freely available on the Web sites of PLoS Medicine at http://www.plosmedicine.org/, Annals of Internal Medicine at http://www.annals.org/, and Epidemiology at http://www.epidem.com/). Information on the STROBE Initiative is available at http://www.strobe-statement.org.
